# Asymptomatic COVID-19 in the elderly: dementia and viral clearance as risk factors for disease progression.

**DOI:** 10.12688/gatesopenres.13357.1

**Published:** 2021-08-27

**Authors:** Ignacio Esteban, Georgina Bergero, Camila Alves, Micaela Bronstein, Valeria Ziegler, Cristian Wood, Mauricio T. Caballero, Diego Wappner, Romina Libster, Gonzalo Perez Marc, Fernando P. Polack

**Affiliations:** 1 INFANT Foundation, Buenos Aires, Argentina; 2 Hospital Militar Central, Buenos Aires, Argentina; 3 Consejo Nacional de Investigaciones Científicas y Técnicas (CONICET), Buenos Aires, Argentina; 4 iTrials, Buenos Aires, Argentina; 5 Swiss Medical Group, Buenos Aires, Argentina

**Keywords:** asymptomatic, pre-symptomatic, elders, COVID-19, risk factors, dementia, geriatric institutions, long-term facilities

## Abstract

Background:

SARS-CoV-2 infected individuals ≥60 years old have the highest hospitalization rates and represent >80% fatalities. Within this population, those in long-term facilities represent >50% of the total COVID-19 related deaths per country. Among those without symptoms, the rate of pre-symptomatic illness is unclear, and potential predictors of progression for symptom development are unknown.

Our objective was to delineate the natural evolution of asymptomatic SARS-CoV-2 infection in elders and identify determinants of progression.

Methods:

We established a medical surveillance team monitoring 63 geriatric institutions. When an index COVID-19 case emerged, we tested all other eligible asymptomatic elders ≥75 or >60 years old with at least 1 comorbidity. SARS-CoV-2 infected elders were followed for 28 days. Disease was diagnosed when any COVID-19 manifestation occurred. SARS-CoV-2 load at enrollment, shedding on day 15, and antibody responses were also studied.

Results:

After 28 days of follow-up, 74/113(65%) SARS-CoV-2-infected elders remained asymptomatic. 21/39(54%) pre-symptomatic patients developed hypoxemia and ten pre-symptomatic patients died(median day 13.5,IQR 12).

Dementia was the only clinical risk factor associated with disease(OR 2.41(95%CI=1.08, 5.39). In a multivariable logistic regression model, dementia remained as a risk factor for COVID-19 severe disease. Furthermore, dementia status showed a statistically significant different trend when assessing the cumulative probability of developing COVID-19 symptoms(log-rank p=0.027).

On day 15, SARS-CoV-2 was detectable in 30% of the asymptomatic group while in 61% of the pre-symptomatic(p=0.012).

No differences were observed among groups in RT-PCR mean cycle threshold at enrollment(p=0.391) and in the rates of antibody seropositivity(IgM and IgG against SARS-CoV-2 nucleocapsid protein).

Conclusions:

In summary, 2/3 of our cohort of SARS-CoV-2 infected elders from vulnerable communities in Argentina remained asymptomatic after 28 days of follow-up with high mortality among those developing symptoms. Dementia and persistent SARS-CoV-2 shedding were associated with progression from asymptomatic to symptomatic infection.

## Introduction

Coronavirus disease 2019 (COVID-19) is particularly severe in the elderly ^1^. SARS-CoV-2 infected individuals ≥60 years of age have the highest hospitalization rates and represent >80% fatalities ^1– 3^. Within this population, those who reside in long-term facilities may represent >50% of the total COVID-19 related deaths per country ^4– 6^.

However, most infected seniors remain asymptomatic and never progress to experience severe disease. While in symptomatic COVID-19 elders, the predisposing risk factors for severe disease are already well described ^2, 3, 7^, among those without symptoms, the rate of pre-symptomatic illness is unclear, and potential predictors of progression for symptom development are unknown.

Our objective was to delineate the natural evolution of asymptomatic SARS-CoV-2 infection and identify determinants of progression to symptomatic illness. For this purpose, we established a prospective cohort of asymptomatic, SARS-CoV-2 infected individuals ≥60 years of age in geriatric institutions and investigated the role of baseline comorbidities, viral load on presentation, viral clearance, and antibody production in disease progression.

## Methods

### Study population

Our group established a medical surveillance team monitoring 63 geriatric institutions in Buenos Aires city and state between June and July 2020. When an index COVID-19 case emerged in one of these residencies, we tested all other consenting, eligible asymptomatic elders ≥60 years old for SARS-CoV-2. Participating seniors were asymptomatic individuals ≥75 years of age, or between 60–74 years with ≥1 comorbidity (hypertension, dementia, diabetes, obesity, chronic renal failure, and/or chronic obstructive pulmonary disease [COPD]).

Institutional review board approval was obtained and all patients or a responsible first-degree family member signed informed consent for their participation in the protocol (Centro de Estudios Infectológicos SA CEIC, Ethics Approval Number 1146).

### Clinical monitoring

SARS-CoV-2 infected, asymptomatic elders were followed daily for 28 days by a medical team using pre-designed questionnaires. Symptoms of COVID-19 included fever (axillary temperature >37.5°C), chills, cough, tachypnea (respiratory rate >20 per minute), physician-diagnosed difficulty breathing, hypoxemia (O _2 _sat<93% when breathing room air), myalgia, anorexia, sore throat, dysgeusia, anosmia, diarrhea, vomiting, and rhinorrhea. Disease was diagnosed when any of these manifestations occurred within 14 days of SARS-CoV-2 detection (95% of symptomatic patients) or between 15 and 28 days of persistently positive real-time reverse transcriptase polymerase chain reaction (RT-PCR) results with no other clinical possible explanation. COVID-19 severe disease was defined as oxygen requirement due to hypoxemia. SARS-CoV-2 load at enrollment, shedding on day 15, and antibody responses at the end of study participation were also studied.

### SARS-CoV-2 and antibody testing

SARS-CoV-2 was assayed in nasopharyngeal and oropharyngeal swabs following Center for Disease Control guidelines at enrollment and day 15 of diagnosis ^8^ **.** Samples were stored in 2 ml of normal saline and tested in duplicate by RT-PCR for SARS-CoV-2 (Atila iAMP® COVID-19).

Antibodies were assayed in 10μl of blood using a validated rapid antibody test (monoclonal immunoglobulin M (IgM) and immunoglobulin G (IgG) against SARS-CoV-2 nucleocapsid protein, SD Biosensor®, Korea) 28 days after enrollment because the test’s sensitivity is reported to be higher at 4–5 weeks ^9, 10^. The assay was performed according to the manufacturer´s protocol ^11^.

### Statistical analysis

Baseline comorbidities were reported using descriptive statistics. Differences between asymptomatic and pre-symptomatic participants were compared using the Student t-test and Chi-squared test, where appropriate. Variables with a p-value <=0.2 were defined as candidates for multivariable evaluation. A p<0.05 was considered statistically significant. Progression to symptomatic illness was assessed using the Kaplan-Meier method, with any COVID-19 related symptom as outcome. Stata/SE 13 package for IBM-PC (Stata Corp) was used for analysis and R Core Team (2019) for Figures.

## Results

### Study Population and clinical evolution

Thirteen of 63 (20%) senior homes presented a positive, symptomatic index case during the study period. In these residencies, we swabbed 258 asymptomatic individuals between June 8 and July 3, 2020. 113 asymptomatic SARS-CoV-2-infected elderly participated in the study. Of these, 100/113 were ≥75 years of age, and 13/113 were between 60–74 years with ≥1 comorbidity ( Table 1).

**Table 1.** Determinants of pre-symptomatic COVID-19.

	Asymptomatic (N=75)	Pre-symptomatic (N=38)	OR (CI 95%)	p-value
Clinical and laboratory				
Median age (IQR) - yr	87.7 (11.57)	86.6 (13.7)	0.99 (0.99-1.00)	0.277 ^*^
Male, no. (%)	14 (19)	6 (16)	0·78 (0.27-2.22)	0.64 ^**^
Smoking history, no. (%)	18 (25)	12 (33)	1.47 (0.61-3.53)	0.385 ^**^
Dementia, no. (%)	28 (39)	23 (61)	2.41 (1.08-5.39)	0.03 ^**^
Hypertension, no. (%)	32 (43)	15 (39)	0.82 (0.37-1.81)	0.624 ^**^
Diabetes, no. (%)	12 (16)	4 (10)	0.59 (0.18-1.97)	0.388 ^**^
Cardiovascular disease, no. (%)	15 (20)	8 (20)	1.02 (0.39-2.66)	0.976 ^**^
Obesity, no. (%)	4 (5)	4 (10)	2 (0.47-8.48)	0.339 ^**^
Cancer, no. (%)	4 (5)	3 (8)	1.46 (0.31-6.87)	0.632 ^**^
Chronic liver disease, no. (%)	1 (1)	1 (3)	1.92 (0.12-31.57)	0.642 ^**^
End-stage renal disease, no. (%)	-	2 (5)	-	-
Asthma, no. (%)	-	2 (5)	-	-
Chronic obstructive pulmonary disease, no. (%)	-	3 (8)	-	-

OR= Odds Ratio, CI = confidence interval, ^*^ Student t test, ^** ^Chi-squared test

**Figure 1.**
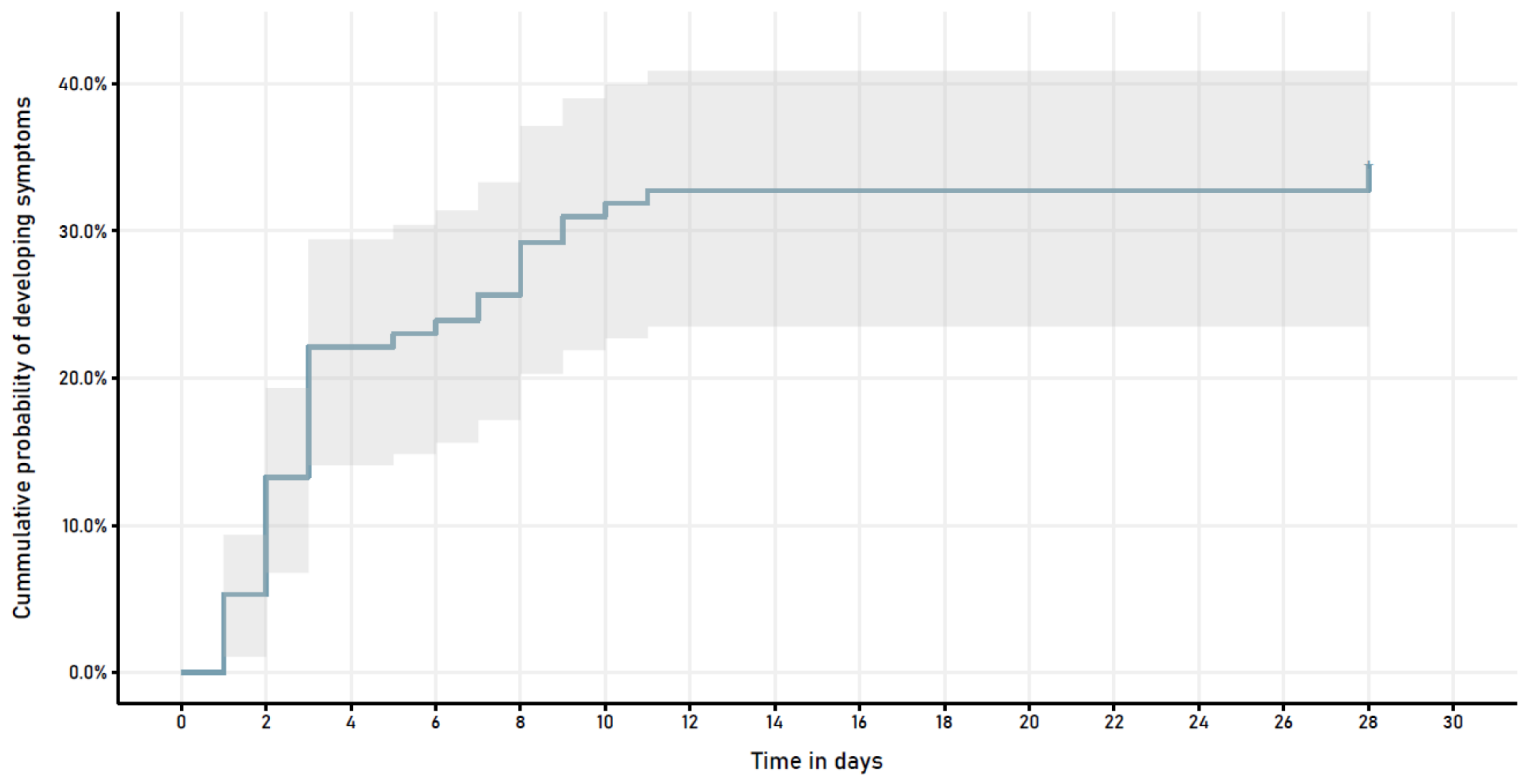
Symptom development in patients who were asymptomatic at time of COVID-19 diagnosis.

Participants' median age was 87 years (IQR 11.85). 93/113 (82%) were females, 98 (87%) had ≥1 comorbidity ( Table 1). After 28 days of follow-up, 74 (65%) elders remained asymptomatic. In 39 (35%) pre-symptomatic patients, the median time to onset of symptoms was 3 days (IQR 6) ( Figure 1). The most frequent presenting symptoms were difficulty breathing (39%), cough (37%), fever (29%), and tachypnea (16%). 21/39 (54%) pre-symptomatic patients developed hypoxemia [21/113 (19%) in the population], a presenting sign in 11/21 (52%). Median time to oxygen supplementation was 4 days (IQR 6); median duration of O2 supplementation in survivors, 4 days (IQR 5). Ten pre-symptomatic patients died (median day 13.5, IQR 12).

### Risk factors for disease progression

None of the baseline conditions classically related to disease severity was associated with symptomatic illness ( Table 1). Dementia was the only clinical risk factor associated with disease (OR 2.41 (95% CI =1.08, 5.39), p=0.03; Table 1) when compared to the asymptomatic patients. These results, with dementia as a potential predictor for the development of symptoms in our population, remained stable in a univariate logistic regression (dementia, p=0.032).

**Figure 2.**
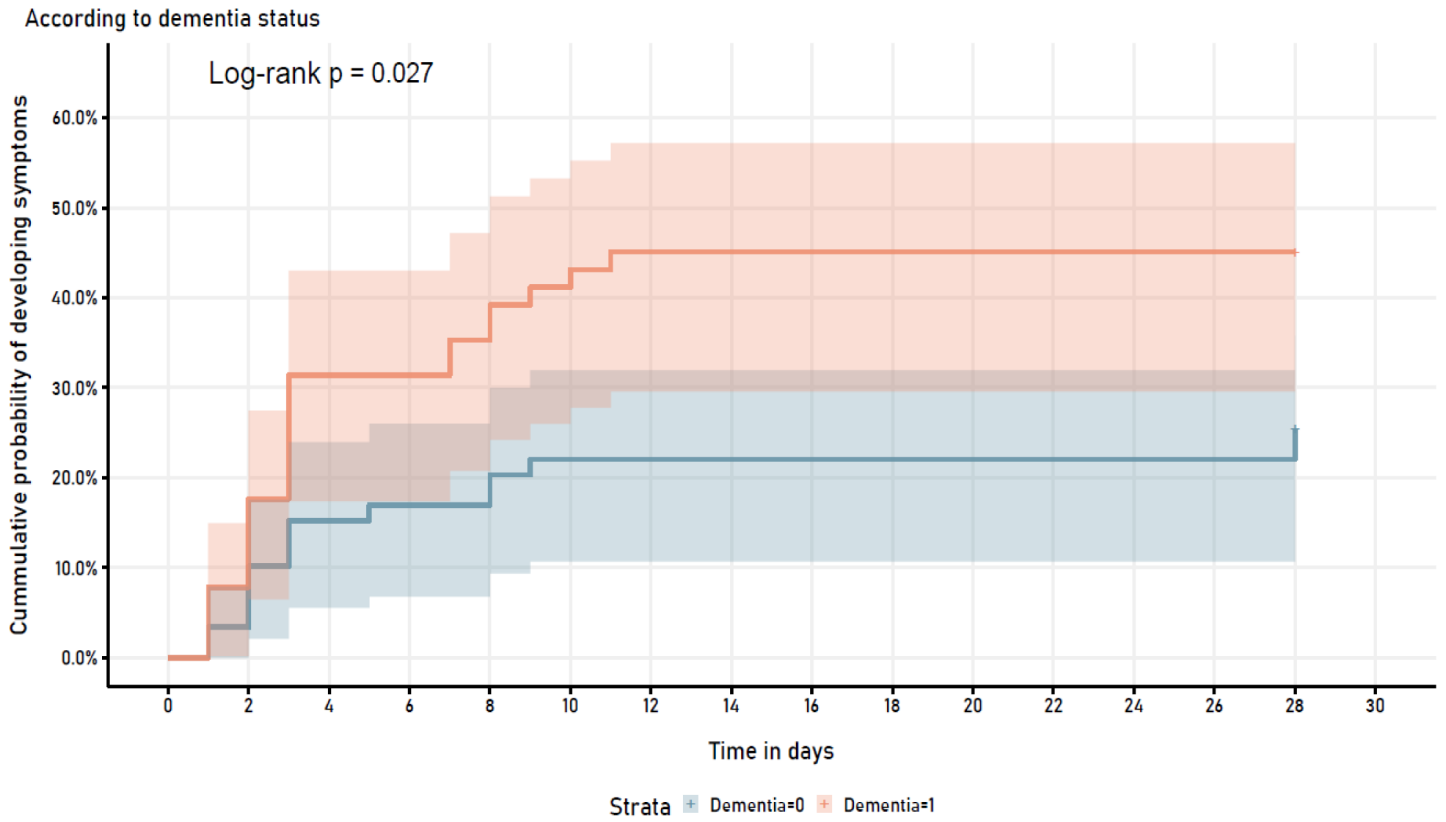
Symptom development in patients who were asymptomatic at time of COVID-19 diagnosis.

 Analyzing potential predictors for COVID-19 severe disease in a multivariable logistic regression model, including variables with a p-value <=0.2 in univariate analysis (cancer history and diabetes), dementia persisted as a risk factor associated with the outcome (OR 3.02 (95% CI =1.08, 8.45), p=0.035) ( Table 2). Furthermore, when assessing the cumulative probability of developing COVID-19 symptoms stratified by dementia diagnosis, it showed a statistically significant different trend in both groups (log-rank p=0.027) ( Figure 2).

**Table 2.** Determinants of COVID-19 severe disease.

	Univariate analysis			Multivariate analysis		
	OR	CI 95%	p-value	OR	CI 95%	p-value
Diabetes	0.26	0.03-2.06	0.2	0.23	0.03-1.9	0.173
Cancer	3.67	0.75-17.81	0.107	4	0.73-21.82	0.11
Dementia	2.81	1.03-7.64	0.043	3.02	1.08-8.45	0.035
Age	1	0.99-1	0.935			
Male	1.6	0.51-5.02	0.419			
Smoking history	1.79	0.66-4.89	0.256			
Hypertension	0.65	0.24-1.76	0.397			
Cardiovascular disease	1.76	0.6-5.21	0.304			
Obesity	1.51	0.28-8.06	0.63			

OR= Odds Ratio, CI = confidence interval

### SARS-CoV-2 viral load and RT-PCR retesting

**Figure 3.**
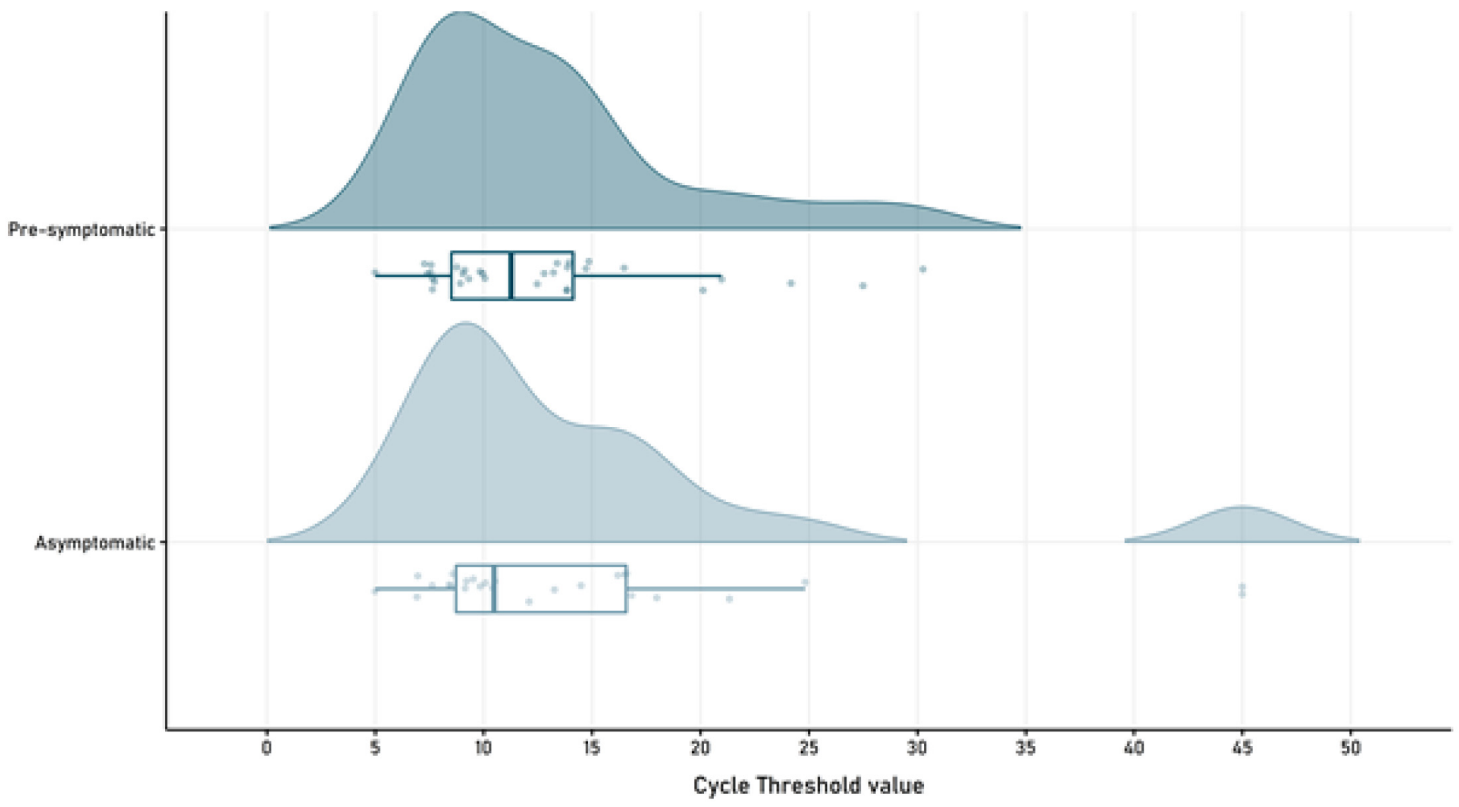
SARS-CoV-2 cycle threshold value in pre-symptomatic and asymptomatic patients.

RT-PCR mean cycle threshold showed no differences among groups at the time of enrollment (p=0.391), with a mean of 14.65 (SD 10.13) in the asymptomatic group and a mean of 12.79 (SD 6.08) in the pre-symptomatic patients ( Figure 3).

 When performing a second RT-PCR testing on day 15 (IQR 1), SARS-CoV-2 was detectable in 30% (14/46) of the asymptomatic subjects, while still present in 61% (17/28) of the pre-symptomatic patients (p=0.012).

### Antibody seropositivity against SARS-CoV-2

All patients were invited to be tested for IgM and IgG against SARS-CoV-2 nucleocapsid protein four weeks after diagnosis. Seventy-six % (77/102) of those alive on day 28 ^th^ of follow-up were assayed with a median day at testing of 28 (IQR 3).

 No differences were observed in the rates of antibody seropositivity between asymptomatic and pre-symptomatic patients respectively (IgM+: 53% (31/59) vs 56% (10/18), p=0.823) (IgG+: 83% (49/59) vs 83% (15/18), p=0.978).

## Discussion

Early recognition of asymptomatic infected patients and defining the determinants of progression from asymptomatic to symptomatic illness in the elderly are critical to examine potential disease-sparing interventions. However, since asymptomatic patients usually do not seek medical assistance or COVID-19 testing, this represents a great challenge.

In our cohort, 2/3 aged study participants remained asymptomatic, only 1/5 developed an oxygen requirement and 9% of all patients died due to COVID-19. Asymptomatic SARS-CoV-2 infected patients play a paramount role in the pandemic, both as sources of viral spreading and as at-risk subjects for hospitalization. Interestingly, few studies examined them in detail ^12^. On the Diamond Princess cruise ship, 88% of asymptomatic and relatively younger patients (median=59.5 years) did not progress to disease ^13^ while, in skilled nursing facilities in the U.S. and in line with our observations, 68% failed to develop illness ^14^. Our case-fatality ratio in symptomatic patients (26%, 10/39), is considerably higher than the one described in Argentina and worldwide ^15^. This emphasizes the relevance of the population under study (residents of long-term facilities), given their high susceptibility for COVID-19 severe disease ^4– 6^.

 Dementia was the sole baseline difference potentially predicting progression to symptomatic disease in our study. Our findings suggest that cognitive impairment plays a key role in disease inception and disease progression in the elderly. Other comorbidities associated with progression from mild to severe symptoms ^2,16^ did not affect the odds of experiencing pre-symptomatic illness in this population. Furthermore, dementia successfully predicted those requiring oxygen. Cognitive impairment has been previously identified in Britain as a risk factor for hospitalization in older patients (OR 3.5 (95% CI =1.93, 6.34)) ^17^. However, our study is the first to prospectively identify dementia as a risk factor for pre-symptomatic illness. There are different reasons behind the elevated mortality seen in long-term facilities worldwide and in our study, that may also explain the role of dementia and cognitive impairment in COVID-19 disease progression. To begin with, residents in geriatric institutions are in close contact with numerous healthcare workers with a consequent increased risk for contracting SARS-CoV-2 infection ^6^. In addition, patients with cognitive impairment may present difficulties in carrying out isolation and the physical distancing needed. Furthermore, patients with dementia were described to present particularly higher blood levels of urea, white blood cell count, and an association with neurological consequences of COVID-19 ^6, 18^.

 No difference in viral load in respiratory secretions was evident at diagnosis between groups, in line with previous reports ^19– 21^. But two weeks after enrollment, the pre-symptomatic group doubled the asymptomatic subjects in the persistence of SARS-CoV-2 detection in respiratory secretions. This longer viral shedding associated with evolution to symptomatic disease, suggests that control of viral replication may influence symptom inception. In line with our findings, infectivity may be weaker in asymptomatic elders than in those fully developing symptoms ^22^. A recent study showed similar results in PCR retesting in SARS-CoV-2 asymptomatic and symptomatic patients, and interestingly, this could be evidenced during the first week after diagnosis ^23^.

Antibody diagnostics tests are critical for detecting asymptomatic patients ^24^. IgM antibodies peak 4 for days after onset of symptoms, declining to become undetectable after 4 weeks. Whereas IgG reaches detectable levels at day 7 and remains highly elevated until 8 months of diagnosis even in asymptomatic patients ^25– 27^. Interestingly, Grossberg *et al.* showed that symptomatic individuals experience a different immune response than asymptomatic SARS-CoV infected patients, revealed by higher levels of IgG against spike 1 and 2 glycoprotein, receptor-binding domain (RBD), and nucleoprotein. While asymptomatic patients may present a more robust IgM response ^28^. 

Nevertheless, IgM and IgG responses were similar with and without symptoms in our cohort, findings aligned with their preventive role in early stages after or even before infection but their lesser influence once disease course has been established ^29^.

Our study has limitations. First of all, older adults, and in particular those with cognitive impairment may present greater difficulties in referring their symptoms. However, all patients were under strictly daily control by nurses and the institution's medical team that accurately reported all symptoms and signs presented. In addition, while no difference was seen among groups in the IgM and IgG levels against SARS-CoV-2 nucleocapsid protein, a more complex analysis of the immune response including neutralizing antibodies, and antibodies titers against spike glycoproteins and RBD, may elucidate differences between groups.

In summary, we present a cohort of SARS-CoV-2 infected elders from vulnerable communities in Argentina, where 2/3 of them remained asymptomatic after 28 days of follow-up with high mortality among those developing symptoms. Dementia and persistent SARS-CoV-2 shedding were associated with progression from asymptomatic to symptomatic infection. Evidently, COVID-19 risk control and prevention are imperative in this high-risk population. These observations may alter our thinking of SARS-CoV-2 asymptomatic infection in the elderly, and if confirmed in other studies, require us to include patients with dementia as candidates for prevention strategies.

## Data availability

Figshare. “Asymptomatic COVID-19 in the elderly: dementia and viral clearance as risk factors for disease progression”. SAS Dataset. DOI: https://doi.org/10.6084/m9.figshare.15050217.v1 ^30^

Figshare. “Asymptomatic COVID-19 in the elderly: dementia and viral clearance as risk factors for disease progression”. Stata dataset. DOI: https://doi.org/10.6084/m9.figshare.15050223.v1 ^31^

Figshare. “Asymptomatic COVID-19 in the elderly: dementia and viral clearance as risk factors for disease progression”. Stata Dofile. DOI: https://doi.org/10.6084/m9.figshare.15050220.v1 ^32^

Data are available under the terms of the Creative Commons Attribution 4.0 International license (CC-BY 4.0).

## References

[1] Garg S Kim L Whitaker M : Hospitalization Rates and Characteristics of Patients Hospitalized with Laboratory-Confirmed Coronavirus Disease 2019 — COVID-NET, 14 States, March 1-30, 2020. *MMWR Morb Mortal Wkly Rep.* 2020;69(15):458–464. 10.15585/mmwr.mm6915e3 32298251 PMC7755063

[2] Wortham JM Lee JT Althomsons S : Characteristics of Persons Who Died with COVID-19 — United States, February 12-May18, 2020. *MMWR Morb Mortal Wkly Rep.* 2020;69(28):923–929. 10.15585/mmwr.mm6928e1 32673298

[3] Li J Huang DQ Zou B : Epidemiology of COVID-19: A Systematic Review and Meta-analysis of Clinical Characteristics, Risk factors, and Outcomes. *J Med Virol.* 2021;93(3):1449–1458. 10.1002/jmv.26424 32790106 PMC7436673

[4] Lau-Ng R Caruso LB Perls TT : COVID-19 deaths in long-term care facilities: a critical piece of the pandemic puzzle. *J Am Geriatr Soc.* 2020;68(9):1895–1898. 10.1111/jgs.16669 32501537 PMC7300761

[5] Fisman DN Bogoch I Lapointe-shaw L : Risk Factors Associated With Mortality Among Residents With Coronavirus Disease 2019 (COVID-19) in Long-term Care Facilities in Ontario, Canada. *JAMA Netw Open.* 2020;3(7):e2015957. 10.1001/jamanetworkopen.2020.15957 32697325 PMC7376390

[6] Livingston G Rostamipour H Gallagher P : Prevalence, management, and outcomes of SARS-CoV-2 infections in older people and those with dementia in mental health wards in London, UK: a retrospective observational study. *Lancet Psychiatry.* 2020;7(12):1054–1063. 10.1016/S2215-0366(20)30434-X 33031760 PMC7535621

[7] Recinella G Marasco G Serafini G : Prognostic role of nutritional status in elderly patients hospitalized for COVID-19: a monocentric study. *Aging Clin Exp Res.* 2020;32(12):2695–2701. 10.1007/s40520-020-01727-5 33034016 PMC7543671

[8] Centers for Disease Control and Prevention: Interim Guidelines for Collecting, Handling, and Testing Clinical Specimens for COVID-19.2020. Reference Source

[9] Jj D Dinnes J Takwoingi Y : Antibody tests for identification of current and past infection with SARS-CoV-2.(Review). *Cochrane Database Syst Rev.* 2020;6(6):CD013652. 10.1002/14651858.CD013652 32584464 PMC7387103

[10] Paiva KJ Grisson RD Chan PA : Validation and performance comparison of three SARS-CoV-2 antibody assays. *J Med Virol.* 2021;93(2):916–923. 10.1002/jmv.26341 32710669

[11] Biosensor SD Standardtm Q : COVID-19 IgM/IgG Duo Test package insert.Gyeonggi-do, Korea; 2020.

[12] He J Guo Y Mao R : Proportion of asymptomatic coronavirus disease 2019: A systematic review and meta-analysis. *J Med Virol.* 2021;93(2):820–830. 10.1002/jmv.26326 32691881 PMC7404334

[13] Sakurai A Sasaki T Kato S : Natural History of Asymptomatic SARS-CoV-2 Infection. *N Engl J Med.* 2020;383(9):885–886. 10.1056/NEJMc2013020 32530584 PMC7304419

[14] White EM Santostefano CM Feifer R : Asymptomatic and Presymptomatic Severe Acute Respiratory Syndrome Coronavirus 2 Infection Rates in a Multistate Sample of Skilled Nursing Facilities. *JAMA Intern Med.* 2020;180(12):1709–11. 10.1001/jamainternmed.2020.5664 33074318 PMC7573793

[15] Mortality Analyses.Johns Hopkins Coronavirus Resource Center. 2021; [cited 2021 Jun 15]. Reference Source

[16] Ejaz H Alsrhani A Zafar A : COVID-19 and comorbidities: Deleterious impact on infected patients. *J Infect Public Health.* 2020;13(12):1933–1839. 10.1016/j.jiph.2020.07.014 32788073 PMC7402107

[17] Atkins JL Masoli JAH Delgado J : Preexisting Comorbidities Predicting COVID-19 and Mortality in the UK Biobank Community Cohort.Editor ’ s choice. *J Gerontol A Biol Sci Med Sci.* 2020;75(11):2224–30. 10.1093/gerona/glaa183 32687551 PMC7454409

[18] Wan Y Wu J Ni L : Prognosis analysis of patients with mental disorders with COVID-19: a single-center retrospective study. *Aging (Albany NY).* 2020;12(12):11238–44. 10.18632/aging.103371 32561692 PMC7343444

[19] Lee S Kim T Lee E : Clinical Course and Molecular Viral Shedding Among Asymptomatic and Symptomatic Patients With SARS-CoV-2 Infection in a Community Treatment Center in the Republic of Korea. *JAMA Intern Med.* 2020;180(11):1447–52. 10.1001/jamainternmed.2020.3862 32780793 PMC7411944

[20] Yu C Zhou M Liu Y : Characteristics of asymptomatic COVID-19 infection and progression: A multicenter, retrospective study. *Virulence.* 2020;11(1):1006–14. 10.1080/21505594.2020.1802194 32722990 PMC7550018

[21] Gao Z Xu Y Sun C : A systematic review of asymptomatic infections with COVID-19. *J Microbiol Immunol Infect.* 2021;54(1):12–6. 10.1016/j.jmii.2020.05.001 32425996 PMC7227597

[22] Gao M Yang L Chen X : A study on infectivity of asymptomatic SARS-CoV-2 carriers. *Respir Med.* 2020;169:106026. 10.1016/j.rmed.2020.106026 32513410 PMC7219423

[23] Al-Rifai RH Acuna J Al Hossany FI : Epidemiological characterization of symptomatic and asymptomatic COVID-19 cases and positivity in subsequent RT-PCR tests in the United Arab Emirates. *PLoS One.* 2021;16(12):e0246903. 10.1371/journal.pone.0246903 33577582 PMC7880695

[24] Long QX Liu BZ Deng HJ : Antibody responses to SARS-CoV-2 in patients with COVID-19. *Nat Med.* 2020;26(6):845–848. 10.1038/s41591-020-0897-1 32350462

[25] Hartley GE Edwards ESJ Aui PM : Rapid generation of durable B cell memory to SARS-CoV-2 spike and nucleocapsid proteins in COVID-19 and convalescence. *Sci Immunol.* 2020;5(54):eabf8891. 10.1126/sciimmunol.abf8891 33443036 PMC7877496

[26] Choe PG Kim KH Kang CK : Antibody Responses 8 Months after Asymptomatic or Mild SARS-CoV-2 Infection. *Emerg Infect Dis.* 2021;27(3):928–931. 10.3201/eid2703.204543 33350923 PMC7920668

[27] Liu X Wang J Xu X : Patterns of IgG and IgM antibody response in COVID-19 patients. *Emerg Microbes Infect.* 2020;9(1):1269–74. 10.1080/22221751.2020.1773324 32515684 PMC7448841

[28] Grossberg AN Koza LA Ledreux A : A multiplex chemiluminescent immunoassay for serological profiling of COVID-19-positive symptomatic and asymptomatic patients. *Nat Commun.* 2021;12(1):740. 10.1038/s41467-021-21040-7 33531472 PMC7854643

[29] Libster R Perez Marc G Wappner D : Early High-Titer Plasma Therapy to Prevent Severe Covid-19 in Older Adults. *N Engl J Med.* 2021;384(7):610–618. 10.1056/NEJMoa2033700 33406353 PMC7793608

[30] Esteban I Bergero G Alves C : “Asymptomatic COVID-19 in the elderly: dementia and viral clearance as risk factors for disease progression”. SAS Dataset. *figshare.* Dataset. 2021. 10.6084/m9.figshare.15050217.v1 PMC8987012 35441127

[31] Esteban I : “Asymptomatic COVID-19 in the elderly: dementia and viral clearance as risk factors for disease progression”. Stata dataset. *figshare.* Dataset. 2021. 10.6084/m9.figshare.15050223.v1 PMC8987012 35441127

[32] Esteban I : “Asymptomatic COVID-19 in the elderly: dementia and viral clearance as risk factors for disease progression”. Stata Dofile. *figshare.* Dataset. 2021. 10.6084/m9.figshare.15050220.v1 PMC8987012 35441127

