# Supplementary material for: Asymptomatic COVID-19 in the elderly: dementia and viral clearance as risk factors for disease progression
Source: Gates Open Res. 2022 Apr 4;5:143. Originally published 2021 Aug 27. [Version 2] doi: 10.12688/gatesopenres.13357.2 (PMC8987012; doi:10.12688/gatesopenres.13357.2)
Supplement: Peer review history

# Peer review: Reviewer response for version 2

**DOI:** 10.21956/gatesopenres.14879.r31915

**Published:** 2022-04-06

**Authors:** Kirsten E. Wiens

**Affiliations:** 1 Department of Epidemiology, Johns Hopkins Bloomberg School of Public Health, Baltimore, MD, USA

The authors have addressed my comments and I have no further comments to make.

Is the work clearly and accurately presented and does it cite the current literature?

Yes

If applicable, is the statistical analysis and its interpretation appropriate?

Partly

Are all the source data underlying the results available to ensure full reproducibility?

Yes

Is the study design appropriate and is the work technically sound?

Yes

Are the conclusions drawn adequately supported by the results?

Partly

Are sufficient details of methods and analysis provided to allow replication by others?

Yes

Reviewer Expertise:

infectious disease epidemiology

I confirm that I have read this submission and believe that I have an appropriate level of expertise to confirm that it is of an acceptable scientific standard.

# Peer review: Reviewer response for version 1

**DOI:** 10.21956/gatesopenres.14601.r31683

**Published:** 2022-02-16

**Authors:** Kirsten E. Wiens

**Affiliations:** 1 Department of Epidemiology, Johns Hopkins Bloomberg School of Public Health, Baltimore, MD, USA

In this paper, Esteban *et al.* conduct a prospective study of 113 SARS-CoV-2-infected elderly adults in 13 senior homes in Buenos Aires during June-July 2020. They follow the study participants over 28 days, monitoring symptoms, viral load, and IgM/IgG responses to SARS-CoV-2 nucleocapsid. They find that the only risk factor significantly associated with developing symptoms following infection was dementia.

 This is an important paper, and one of its strengths is that the study team prospectively monitored 63 senior homes in Buenos Aires for symptomatic index cases in order to identify participants for inclusion, allowing them to actively recruit asymptomatically infected individuals whether or not they sought testing. There are also a few components of the analysis and discussion of the results that could be improved:

1. Throughout the manuscript, I’d suggest being careful with the use of the terms “determinants” and “was predictive of”. The authors have shown that dementia is strongly associated with increased risk of developing symptoms among the elderly, but they don’t actually test how good the different risk factors are in prediction (e.g., through cross-validation).
2. It would be helpful to have further discussion and/or analysis of additional variables that may be associated with symptoms. For example, were there differences in severity or extent of outbreaks in the senior homes (which might impact viral dose or number of exposures)? If so, these could be examined in the regression models and/or senior home could be included as a random effect. Various other individual-level factors may impact severity (nutrition, past infection, exercise, etc), which the authors may not be able to evaluate, but could discuss.
3. Why was the multivariable logistic regression only run for severe COVID-19 disease, rather than for asymptomatic vs. pre-symptomatic? For consistency with the rest of the paper, I think it’s important to include this analysis for symptomatic disease.
4. Why was a p value of 0.2 used to select covariates in the multivariable logistic regression? What happened if you put all the covariates in? Or just remove highly collinear covariates?

Minor comments:

- I suggest including the study location (Bueno Aires, Argentina) and dates (June-July 2020) in the methods of the abstract. 
- 258 asymptomatic individuals were swabbed and 113 asymptomatic SARS-CoV-2 infected individuals were included. How many of the 258 were positive but declined to participate or were lost to follow-up?
- Dementia was also the most common comorbidity in this cohort. The authors might speculate on whether their findings may be different in larger cohorts or populations where risk factors such as obesity or cancer are more common.
- In the first sentence of the discussion, I’d use percentages rather than fractions (so that those denominators don’t get confused with sample sizes).

Is the work clearly and accurately presented and does it cite the current literature?

Yes

If applicable, is the statistical analysis and its interpretation appropriate?

Partly

Are all the source data underlying the results available to ensure full reproducibility?

Yes

Is the study design appropriate and is the work technically sound?

Yes

Are the conclusions drawn adequately supported by the results?

Partly

Are sufficient details of methods and analysis provided to allow replication by others?

Yes

Reviewer Expertise:

infectious disease epidemiology

I confirm that I have read this submission and believe that I have an appropriate level of expertise to confirm that it is of an acceptable scientific standard, however I have significant reservations, as outlined above.

# Response

**Published:** 2022-03-26

**Authors:** Ignacio Esteban

**Affiliations:** INFANT Foundation, Buenos Aires, Argentina

Response to Reviewer 2:

 In this paper, Esteban et al. conduct a prospective study of 113 SARS-CoV-2-infected elderly adults in 13 senior homes in Buenos Aires during June-July 2020. They follow the study participants over 28 days, monitoring symptoms, viral load, and IgM/IgG responses to SARS-CoV-2 nucleocapsid. They find that the only risk factor significantly associated with developing symptoms following infection was dementia.

 This is an important paper, and one of its strengths is that the study team prospectively monitored 63 senior homes in Buenos Aires for symptomatic index cases in order to identify participants for inclusion, allowing them to actively recruit asymptomatically infected individuals whether or not they sought testing. There are also a few components of the analysis and discussion of the results that could be improved:

**We greatly appreciate Reviewer 2’s perspective and input. We have made modifications to the article addressing these topics. Please find specific comments to each commentary below.**

- Throughout the manuscript, I’d suggest being careful with the use of the terms “determinants” and “was predictive of”. The authors have shown that dementia is strongly associated with increased risk of developing symptoms among the elderly, but they don’t actually test how good the different risk factors are in prediction (e.g., through cross-validation).

**We thank the reviewer for this comment. We have addressed this through the article accordingly. In addition, we have added it as a study limitation in the discussion section.**

  

- It would be helpful to have further discussion and/or analysis of additional variables that may be associated with symptoms. For example, were there differences in severity or extent of outbreaks in the senior homes (which might impact viral dose or number of exposures)? If so, these could be examined in the regression models and/or senior homes could be included as a random effect. Various other individual-level factors may impact severity (nutrition, past infection, exercise, etc), which the authors may not be able to evaluate but could discuss.

**The reviewer is correct. There are other variables such as nutrition status, exercise, and frailty that could also explain the effect of dementia on the development of symptoms and COVID-19 severe disease.  We have addressed these now in our discussion section since data for those variables was not available at the time. None of the participants were previously infected with SARS-CoV-2.**

**To evaluate the potential effect of the number of infected patients per senior home we included a new variable according to the distribution of our participants in each senior home to compare this, which showed no association with the outcomes nor confounding on the relationship between dementia and the outcomes of interest. This information was now added to the results section, Table I and Table II, and is now available in the updated dataset.**

  

- Why was the multivariable logistic regression only run for severe COVID-19 disease, rather than for asymptomatic vs. pre-symptomatic? For consistency with the rest of the paper, I think it’s important to include this analysis for symptomatic disease.

**We thank the reviewer for this comment since we agree it is key for the correct interpretation of the results. For the first analysis, seeking for potential determinants associated with the status pre-symptomatic we only ran a univariate analysis since as we mentioned in the Statistical Analysis section and the reviewer in her next comment, we only selected co-variates with a p-value <=0.2 on univariate analysis. Given our sample size and the number of events per outcome (pre-symptomatic disease = 39, COVID-19 severe disease = 21) we selected this previously validated strategy to conduct a purposeful selection of covariates ^1,2^. Given that none of the studied co-variates reached this threshold we did not conduct a multivariable analysis for this outcome.**

**Nevertheless, we agree a multivariate analysis may strengthen our analysis and we thank the reviewer for this comment. We have now added this new information, a multivariate analysis in table I and in the Results section.**

  

- Why was a p value of 0.2 used to select covariates in the multivariable logistic regression? What happened if you put all the covariates in? Or just remove highly collinear covariates?

**As per the comment above, we have now added all of the discussed variables to the multivariable analysis where the potential association between dementia and COVID-19 severe disease remained (please find this in the Results section and in Table II).**

 Minor comments:

- I suggest including the study location (Bueno Aires, Argentina) and dates (June-July 2020) in the methods of the abstract. 

**       We have now added this information to the abstract.**

  

- 258 asymptomatic individuals were swabbed and 113 asymptomatic SARS-CoV-2 infected individuals were included. How many of the 258 were positive but declined to participate or were lost to follow-up?

**All of the participants with a positive result on the nasal swab accepted to participate in the study. The remaining 145 participants that were not included in the study had a negative COVID-19 test. We have clarified this information in the Results section.**

**None of the patients was classified as loss to follow-up, due to the established surveillance strategy, the size of our cohort, and the follow-up time.**

  

- Dementia was also the most common comorbidity in this cohort. The authors might speculate on whether their findings may be different in larger cohorts or populations where risk factors such as obesity or cancer are more common.

**The reviewer is correct. We have addressed this now in the Discussion section.**

  

- In the first sentence of the discussion, I’d use percentages rather than fractions (so that those denominators don’t get confused with sample sizes).

**We thank the reviewer for this comment. We have modified this sentence in the Discussion section.**

****

**References:**

 1. Bendel R, Afifi A. Comparison of Stopping Rules in Forward “Stepwise” Regression. *J Am Stat Assoc*. Published online 1977:37-41. doi:http://dx.doi.org/10.1080/01621459.1977.10479905

 2. Greenland S, Mickey RM. The impact of confounder selection criteria on effect estimation. *Am J Epidemiol*. 1989;130(5):1066. doi:10.1093/oxfordjournals.aje.a115409

# Peer review: Reviewer response for version 1

**DOI:** 10.21956/gatesopenres.14601.r31188

**Published:** 2021-10-06

**Authors:** Carlota Dobaño

**Affiliations:** 1 ISGlobal, Hospital Clínic, University of Barcelona, Barcelona, Spain

The article by Esteban *et al.* presents a longitudinal study in long-term geriatric facilities to evaluate the proportion of individuals with asymptomatic vs symptomatic SARS-CoV-2 infection and the risk factors for negative disease progression and mortality in Argentina. Data from multivariable models indicate that dementia and persisting viral shedding were associated with negative outcomes in the elderly. This is a well done and valuable study to better understand the rate of pre-symptomatic illness and prognosis of COVID-19 when still asymptomatic, in one of the most vulnerable populations in an urban environment.

 Some comments:

- "those in long-term facilities represent >50% of the total COVID-19 related deaths per country." Indicate what country or what type of countries as this may not be universal worldwide.
- Among limitations, it could be that the other comorbidities studied that were not significant predictors could have a role in larger studies (hypertension, diabetes, obesity, chronic renal failure, COPD). Please discuss.
- Antibody data was only analyzed as categorical variable (seropositive-seronegative) with rapid tests, but there could be a value to look into magnitude of response in relation to outcomes. Did authors attempt this analysis or do they have samples stored for antibody or biomarker analyses?. As indicated, "a more complex analysis of the immune response including neutralizing antibodies, and antibodies titers against spike glycoproteins and RBD, may elucidate differences between groups."
- For interpretation: the finding that dementia was associated with negative disease progression. Beyond cognitive impairment, distancing, urea and FBC that are mentioned. Since the physiological causes of dementia are not fully understood, could there be immunological basis that might be shared with mechanisms that have been linked to severe COVID-19? (e.g. immune senescence and unbalanced inflammation phenomena). Could some discussion elude to this option?
- Similarly, the inability to control the virus in the mucosa (persistent PCR+ being also associated with outcomes) could be linked to immunological dysfunction in those patients? This could also be briefly discussed.

Is the work clearly and accurately presented and does it cite the current literature?

Yes

If applicable, is the statistical analysis and its interpretation appropriate?

Yes

Are all the source data underlying the results available to ensure full reproducibility?

Yes

Is the study design appropriate and is the work technically sound?

Yes

Are the conclusions drawn adequately supported by the results?

Yes

Are sufficient details of methods and analysis provided to allow replication by others?

Yes

Reviewer Expertise:

Immunology, vaccines, infectious diseases, global health

I confirm that I have read this submission and believe that I have an appropriate level of expertise to confirm that it is of an acceptable scientific standard.
